# Driving Activity Recognition Using UWB Radar and Deep Neural Networks

**DOI:** 10.3390/s23020818

**Published:** 2023-01-10

**Authors:** Iuliia Brishtel, Stephan Krauss, Mahdi Chamseddine, Jason Raphael Rambach, Didier Stricker

**Affiliations:** 1 Department of Augmented Vision, German Research Center for Artificial Intelligence, Trippstadter Str. 122, 67663 Kaiserslautern, Germany; 2 Department of Computer Science, RPTU, Erwin-Schrödinger-Str. 57, 67663 Kaiserslautern, Germany

**Keywords:** modern radar applications, artificial intelligence and machine learning for radar, radar sensors for driver monitoring, radar signal processing techniques

## Abstract

In-car activity monitoring is a key enabler of various automotive safety functions. Existing approaches are largely based on vision systems. Radar, however, can provide a low-cost, privacy-preserving alternative. To this day, such systems based on the radar are not widely researched. In our work, we introduce a novel approach that uses the Doppler signal of an ultra-wideband (UWB) radar as an input to deep neural networks for the classification of driving activities. In contrast to previous work in the domain, we focus on generalization to unseen persons and make a new radar driving activity dataset (*RaDA*) available to the scientific community to encourage comparison and the benchmarking of future methods.

## 1. Introduction

Under the increasing level of automation available in production vehicles, continuous driver monitoring becomes a crucial safety factor [1]. To ensure drivers remain undistracted in the driving loop [2] and to prevent a negative impact of the autonomous system on the ability of drivers to take over [3,4,5], multiple methods from various research fields including human–machine interaction, psychology, computer science, and ergonomics have been investigated. Thus, in-cabin driver monitoring cameras [6,7] and eye-tracking systems were tested [3,8,9,10,11,12] and partly integrated into production vehicles. Each of these technologies has its own strengths and weaknesses. For instance, despite the high precision of distraction recognition [13,14], in-cabin cameras are considered by many drivers as an intrusion into their privacy. Furthermore, eye-tracking systems cannot fully infer the engagement of the drivers in the driving loop even if their eyes are directed on the road [9].

Radar technologies experience a growing interest in the field of human activity recognition (HAR) and human monitoring. Compared to other optical systems, radar provides unrivaled advantages in terms of privacy, robustness to environmental conditions, low sensitivity to obstacles and hazards, as well as usability [15], enlarging the number of potential areas of application. While the main area of radar applications for HAR remains indoor activity classification [4,15,16,17,18,19,20,21,22], vital sign monitoring [23,24,25] and fall detection [26,27], another prospective field is driver monitoring. Several companies have already integrated radar-based solutions for presence and seat occupancy detection [28] as well as vital sign recognition [29,30,31]. Recent studies also point to the feasibility of radar systems to recognize drivers’ behavior and physical state even in moving vehicles using radar [30,32].

Most of the studies deploying radar systems for HAR reported outstanding classification performance of their machine learning models: in some cases, multi-class classifications exceeded an average accuracy of 90% [4,19,20,22,32]. However, a detailed examination of these studies raises several questions regarding the generalization ability of the models. In particular, radar data acquired from multiple persons are commonly split randomly into training and test datasets [4,19,20,22]. As a consequence, data from the same participant can potentially be seen by the model both during training and validation. Taking into account the general ability of radar for biometric authentication [33,34], this technique does not investigate the ability of the model to generalize to new users. Another problem is the limited availability of radar datasets, which is crucial for the reproducibility of reported results.

Addressing the aforementioned safety issues including driver distraction or long-term autopilot utilization, we investigate the feasibility of a low-cost UWB radar for driving activity recognition. In particular, we record six activities associated with conventional, autonomous, and distracted driving. Because of safety issues for the driver and passengers, as well as currently restricted legal utilization of the autopilots under local law, the study was performed under simulated driving conditions. Using a Convolutional Neural Network (CNN) and a Long Short-Term Memory neural network (LSTM), we evaluate the generalization ability of the network by comparing the prevalent practice of random stratified data splitting versus the more strict leave-one-participant-out cross-validation method. We use Doppler data with a simple interquartile range (IQR) normalization method avoiding extensive pre-processing steps (which might heavily depend on the used radar system [35]). This ensures the real-time application capability of the system and enhances the transferability of the method between different radar systems. Finally, in contrast to prior work on the topic, we provide access to the dataset acquired in this study in order to encourage comparison and enable the reproducibility of results. While several radar datasets in HAR and the healthcare domain are available [36,37,38], to the best of our knowledge, so far, there are no public datasets available that contain radar data of driving activities. To summarize, our work introduces the following main contributions:

- We introduce a novel method for normal, autonomous, and distracted driving activity recognition using an ultra-wideband radar and Deep Neural Networks.
- We evaluate the generalization ability of radar-based driving activity recognition to persons not seen in the training data.
- We show that applying an IQR normalization method significantly improves the generalization ability of the neural networks for previously unseen persons.
- We introduce *RaDA*, the first UWB radar dataset for driving activity classification, and make it publicly available to facilitate the comparison and benchmarking of methods in the field.

## 2. Related Work

### 2.1. Radar and Driver Recognition

A wide range of radar types can be found in the field of HAR [15]. The most commonly used types can be divided into two families: Continuous-Wave (CW) and Pulse radars. Because an extensive overview of radar systems is not in the scope of this work, we focus only on the systems that were used in this work and related studies.

Continuous-wave radars continuously transmit radio energy at high frequencies, and the radar echo is received and processed continuously as well. Frequency-Modulated Continuous-Wave (FMCW) radars belong to the CW group and transmit a frequency-modulated electromagnetic wave and capture its scattering from the targets. Based on the properties of the captured scattering, the distance, velocity, size, and orientation of the targets can be calculated [39]. In contrast to CW radars, pulsed radars transmit for a short time followed by a long pause while the radar is in receive mode. Ultra-wideband (UWB) is a family of pulsed radars that transmit low-powered pulses over a wide spectrum [16] allowing them to have a higher range resolution resulting in more fine-graded information about the target [40]. UWB radars are able to resolve the conflict between Doppler and range resolution while capturing the Doppler information of each scattering center of the human body [15]. Moreover, they are robust to multi-path distortion [41] and have a low energy consumption.

Despite the rising interest in radar in the context of in-cabin driver monitoring, the existing work comprises only very few publications. The potential of pulse ultra-wideband radar for in-cabin driver health monitoring and smartphone utilization was demonstrated in a study by Leem et al. [30]. The authors provided a detailed description of pre-processing and reconstruction of the leaking breathing pattern under different driving activities. They also introduced an algorithm to detect drivers’ smartphone usage, pointing at radar technology as a potential technique for preventing car crashes. Similarly, Ding et al. used an FMCW radar for the detection of inattentive driver behavior [32]. The authors run a series of experiments in a real car environment, where the drivers performed seven different activities including head flexion, rotation, and shaking, as well as body movement, sleepy behavior, and picking up a smartphone. Using range–Doppler maps, they extracted a new activity representation called a dynamic-Doppler trajectory (DRDT) map. Then, the associated activities from the DRDT range of interest, Doppler energy change, and dispersion features were extracted and used to build machine learning algorithms. Using decision trees, SVM, KNN and ensemble classifiers, the highest average accuracy they achieved for the task of in-cabin activity classification was 95%. It is important to note that the recorded activities primarily considered head motions, flexion, and rotation.

### 2.2. Radar and Deep Learning

The research on HAR demonstrated outstanding results and multiple advantages of DL techniques for the classification of radar data. In particular, previous studies showed that radar echo data can be treated both as an image in the form of a spectrogram or as time series of the intensity values [15].

Using a pre-trained and fine-tuned ResNet-18 and simulated micro-Doppler spectrograms, Du et al. [42] achieved an average accuracy of 97.92% for six classes including walking, boxing, crawling, jumping, and standing. Shao et al. [20] recorded six participants performing similar actions as in the aforementioned work using a UWB radar. Creating a simple CNN model and using only range information for model training, they reached an average accuracy of 95.24% for activity recognition. However, their validation dataset resulted from a random splitting of the data on the level of individual samples and not participants. Using a dataset with 1633 micro-Doppler spectrograms relating to six classes including falling, Taylor et al. [19] evaluated six different machine learning models. They showed that CNN (in combination with PCA) achieved the highest classification accuracy of 95.30%. Finally, Vandersmissen et al. [39] released two datasets containing gesture and event data captured by an FMCW radar along with a video camera. They evaluated five different modifications of LSTM and CNN networks on 2347 and 1505 samples of six different types of gestures and events (entering and leaving the room, sitting down, standing up, clothe, unclothe). They found that a 3D-ResCNN achieved the lowest error rate of 1.67% in the classification performance of events while random splitting and 2.97% when using leave-one-out cross-validation, respectively. In addition, the authors pointed at the existing opportunity for radar and video data fusion for situations where visual information becomes inaccessible or undesirable.

Considering radar data as time series with time-varying properties, several authors proposed LSTM-based classification approaches for HAR. Using raw spectrograms of six obtained activities (walking, sitting down, standing up, picking up an object, drinking water, and falling), Taylor et al. [19] reported an average accuracy of 80.48% for Uni-LSTM and 83.53% for Bi-LSTM. Noori et al. [22] classified five activities (lying, sitting on the bed with the legs on the bed, sitting on the bed with the legs on the floor, standing, and walking) obtained from 13 participants using a UWB radar. Using an Enhanced Discriminant analysis with LSTM, they achieved an average classification accuracy of 99.6%. However, after applying the leave-one-out cross-validation strategy, the overall classification performance dropped to 66%. Li et al. [43] investigated a bi-directional LSTM approach for HAR. They used six activities (walking, running, jumping, boxing, standing, creeping) from the MOCAP database [44] to build an LSTM model. Their bi-directional LSTM achieved 90.3% accuracy. They also evaluated the impact of the sequence length on the classification performance and found a length between 0.6 and 1 second to be sufficient for the optimal classification performance [43].

Taking these results together, it can be concluded that both visual and time-varying representations of radar data perform on a very high level in human action recognition tasks. At the same time, only a few works [22,39,45] reported results for random stratified data splitting and cross-validation, where a significant difference was observed. Importantly, the aforementioned studies used classes such as regular walking, crawling, standing up, and sitting down, where each action itself has a unique, clearly distinguishable pattern.

## 3. Proposed Approach

As motivated earlier, we use a residual neural network and an LSTM to classify six different driving activities recorded with a UWB radar. The radar hardware automatically outputs the pulse-Doppler data after internally performing the fast Fourier transforms (FFT) on the time domain samples. We then define the elements of the range-Doppler map *W* as $w_{k,f}\in R$, where *w* denotes the Doppler pulse for a given range bin *k* and Doppler frequency *f*. Range-Doppler maps $W_{t}$ are generated at each frame measurement *t*.

Based on empirical studies that showed two seconds of distracted behavior are sufficient for an increased risk of accidents [9,46], we chose a window size of 1 s. Prior to being processed by deep learning models, the acquired data were cleaned from outliers using the interquartile range (IQR). As outliers, we considered range-Doppler bins with an amplitude exceeding the range: *Q3 + 1.5 ∗ IQR*, where Q3 is the third quartile (or 75th percentile). Excessive amplitude values are caused by strong reflections from metallic objects, e.g., in parts of the car seats. The values exceeding this range were replaced by the maximum value within this range. We selected the IQR method because it automatically adapts to the scale of data and determines cut-off points which are independent of the specific type of radar (e.g., FMCW, UWB). Importantly, the IQR coefficient was calculated on the training data only and was applied for data normalization in training as well as validation.

**Figure 1:**
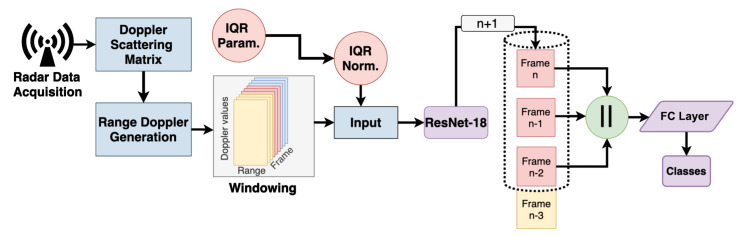
Flow diagram of the inference pipeline of the proposed approach. *n* represents the frame counter. The Doppler data are fed to the ring buffer frame by frame. Three frames, which represent roughly one second, are then concatenated. The concatenated frame data are further forwarded to the fully connected (FC) layer of the network.

The architecture of our ResNet-based approach (see Figure 1) was designed with real-time application in mind. We transform the radar data into spectrograms that represent range-Doppler maps. Three of them cover approximately a time span of 1 s (more details in Section 4.1). Each range-Doppler map is processed independently by the same ResNet-18 to extract features. The features of the last three frames are kept in a ring buffer. This way, whenever a new frame arrives, only this single frame needs to be processed by ResNet. Then, the features of the three frames are concatenated and classified jointly by a fully-connected layer. Training of this architecture is performed through the use of three parallel ResNet-18 instances that share their weights. This ensures the proper flow of the gradients during training and enables training with random shuffling.

## 4. Experimental Setup and Dataset

In this section, we describe the performed experiments for in-cabin driver activity classification. We start with the baseline definition based on the re-implementation of the work of Ding et al. [32]. Then, we investigate the performance of ResNet-18 and an LSTM.

### 4.1. Radar

**Figure 2:**
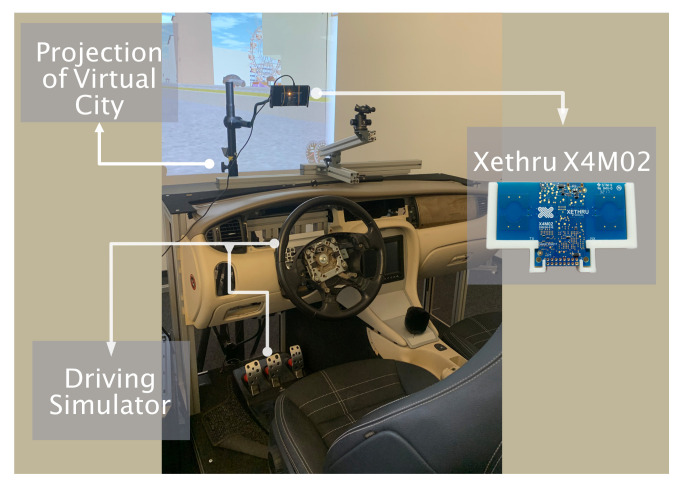
View of the driving simulator recording environment with the UWB Radar Xethru X4M02 in place.

We used the ultra-wideband (UWB) radar sensor X4M02 http://laonuri.techyneeti.com/wp-content/uploads/2019/02/X4M02_DATASHEET.pdf (accessed on 14 November 2022) which can detect and monitor human movements within the operating detection range [25]. Table 1 lists the radar settings used for data recording. The radar placement was carried out following the empirical evaluations of Thullier et al. [47]. The detection zone was set to 0.40 to 1.20 m, and the sensor was placed at a height of 60 cm over the cockpit directed at the center of the driver seat (see Figure 2) to minimize obstacles and interference. This corresponds to a placement of the radar at the top of the windshield in a real car.

We used the library *ModuleConnector* https://github.com/novelda/Legacy-SW (accessed on 14 November 2022) to develop our own script for recording and extraction of radar data. The radar was sampled with an extended frequency of 50 Hz. Because of the internal buffering process http://laonuri.techyneeti.com/wp-content/uploads/2019/02/X4M200_DATASHEET.pdf (accessed on 14 November 2022) of the Xethru radar, the resulting Doppler data had a frequency of 2.9 Hz. We acquired pulse-Doppler data containing the pulse magnitudes for all range bins and range values in the measured domain as well as the Doppler frequencies.

### 4.2. Driving Simulation Environment

The dataset was acquired in a mounted driving simulator which consisted of a Jaguar XJ 4.2 V8 Executive cockpit along with the integrated input controller Logitech G27 Driving Force comprised of a steering wheel, throttle, and brake pedals. To achieve a realistic driving behavior, the highly immersive driving simulation software OpenDS https://opends.dfki.de/ (accessed on 25 July 2021) was used. All driving tasks were performed using an automatic transmission.

### 4.3. RaDA Dataset

**Figure 3:**
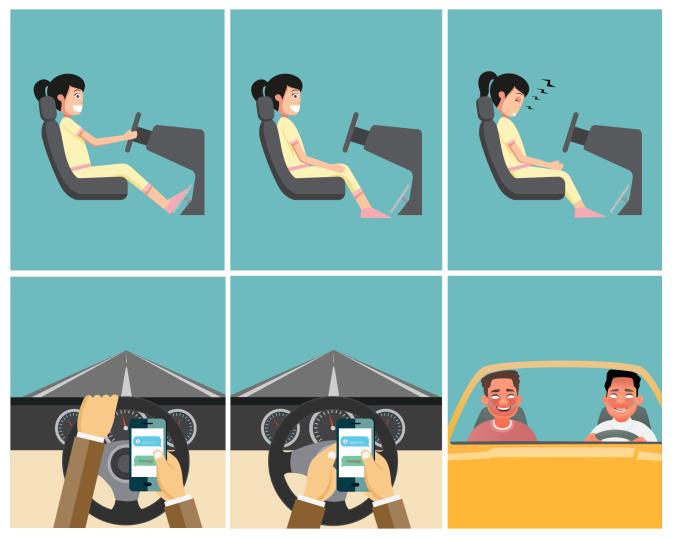
Overview of six driving activities recorded with UWB radar Xethru X4M02. Top (**left** to **right**): Driving, Autopilot, Sleeping. Bottom (**left** to **right**): Driving and Smartphone Utilization, Smartphone Utilization, Talking to Passenger. Credits: Adobe Stock.

Ten participants (one female) were asked to perform six activities as introduced in Table 2 and shown in Figure 3. Each participant performed the activities in the same fixed order. Each activity was recorded separately in a continuous manner. The total recording duration for each activity was set to one minute (small deviations exceeding one minute are possible). Thus, the provided dataset includes approximately 60 min of driving activities. Table 3 provides information about the height and weight of participants included in the dataset.

#### Action Performance Protocol

1. *Autopilot*. While driving with autopilot, participants were instructed to keep their hands on their knees while sitting in the simulator and observing the virtual environment.
2. *Driving*. Participants were asked to drive freely through the virtual city following the general traffic rules. They were also instructed to turn at least once.
3. *Sleeping*. For the sleeping action, participants were asked to take a comfortable position in the driving chair while keeping their head in ventral flexion, close their eyes and relax.
4. *Smartphone utilization*. We used the same instruction as for *autopilot*, with the addition to check e-mails or social media using their smartphone with both hands.
5. *Driving and Smartphone utilization*. During this action, the participants had to perform driving while steering the wheel with the left hand and checking e-mails, social media, etc. using their right hand.
6. *Talking to passenger*. A second person was invited as a passenger to take the front seat. The drivers were instructed to actively communicate with the passenger while rotating their head toward the passenger and using the right hand for gesticulation if preferred. At the same time, they had to perform regular driving.

**Figure 4:**
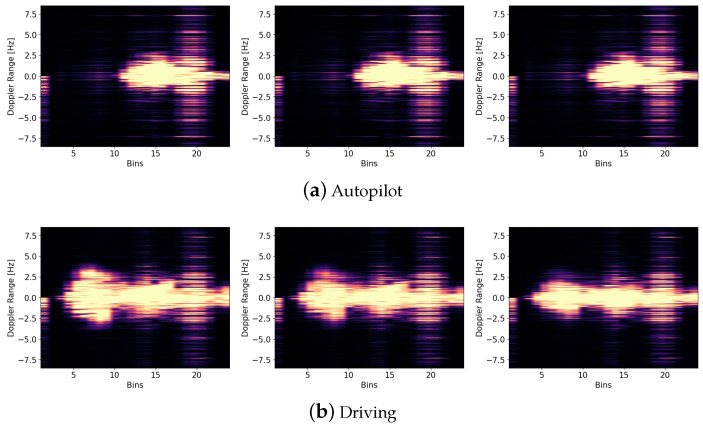

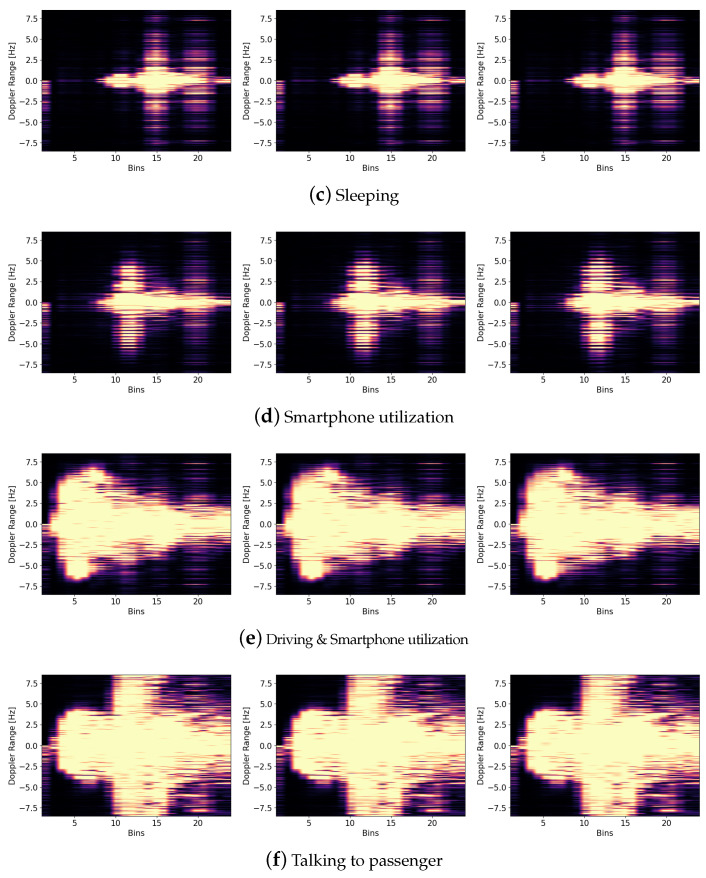
Range-Doppler spectrograms of six (**a**–**f**) in-cabin activities captured by the radar. Three images within one class represent roughly one second.

Figure 4 represents the Doppler spectrograms for each class. Figure 3 provides graphical representations of the six recorded classes. The aforementioned driving activities belong to three driving behaviors: normal driving (driving), autonomous driving (autopilot), and distracted driving (remaining classes), where the classes *sleeping* and *smartphone utilization* are considered as distracted behavior during autonomous driving. The definition of distracted driving behavior was in accordance with [48].

## 5. Experiments and Results

In this section, we describe the performed experiments for driver behavior recognition using range-Doppler maps. We report the results using *Classification Accuracy* (correctly classified activity windows divided by the total number of activity windows), and the F1-score for a better comparison (see Table 4). For the deep learning models, we used the PyTorch library [49], while for the classic machine learning algorithms the scikit-learn library was used.

**Figure 5:**
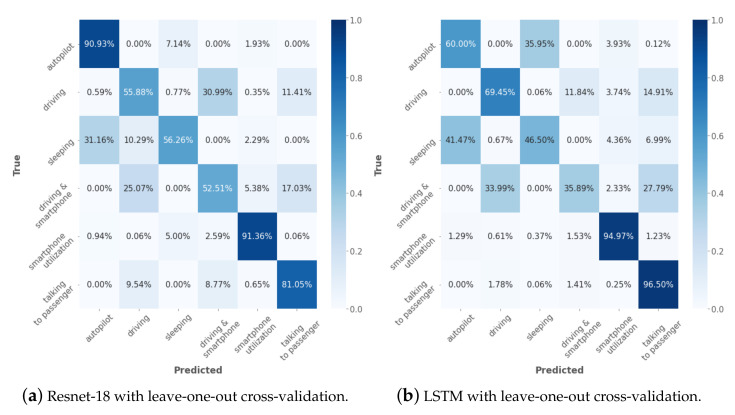
Confusion matrices of obtained classification results using ResNet-18 and LSTM.

We ran two different experiments for data splitting and evaluation. In the first experiment, we used random stratified data splitting for the acquired radar data into training (80%) and test (20%) sets as in studies [4,19,20,22]. This was performed to evaluate the ability of the architecture to overfit on the radar data of specific persons. In the second experiment, we performed leave-one-out cross-validation, where the whole data of one participant were withheld from the training dataset and used for validation only. The cross-validation was repeated 10 times according to the number of participants. The final accuracy is reported as an average value of over ten participants. We additionally provide confusion matrices for the best-performing models (see Figure 5). Importantly, the goal is not to directly compare the classification performance between ResNet and LSTM. Given the difference in the model architectures and the way they treat data, there is no way to provide an honest comparison between them. We rather evaluate the performance of ResNet-18 and LSTM on the given dataset. To examine the scalability of the systems with lower computational power, we calculated the inference time of the ResNet-18 on a Geforce GTX 1080Ti GPU. The resulting inference time was 10.38 ms. The inference for a single sequence using LSTM on an Intel Core i9-9880H CPU took on average 55.5 ms. Both results validate the real-time capability of the approach.

### 5.1. Baseline Classification

**Figure 6:**
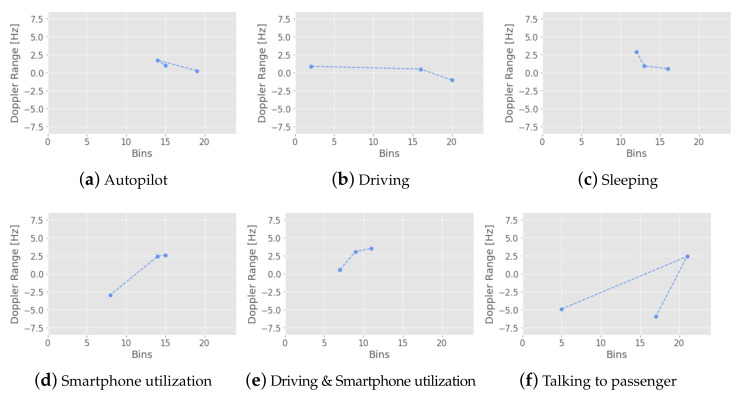
Range-Doppler trajectories of six (**a**–**f**) in-cabin activities calculated using the method of [32]. Each trajectory contains a single frame (0.34 s).

Setting a baseline to compare our method to is challenging due to the very small number of existing radar-based driver monitoring approaches overall, with none providing a source code or a dataset for comparison. Nevertheless, in order to define a baseline, we re-implemented the method proposed by [32] based on the information provided in their paper. The method uses range-Doppler frames and time-Doppler spectrograms obtained from the in-cabin driver recording to generate features. Because of hardware differences, in our implementation, we focused on the features extracted from the range-Doppler trajectory (RDT), in particular, *dynamic Doppler*, *Doppler range* and *dynamic power* because of the similarity to our output data. Among the 12 classifiers evaluated by [32], the ensemble classifier with bagged trees achieved the highest classification accuracy of 93.3% for the range-Doppler trajectory reported on their dataset. We calculated the features on the level of single participants using a window size of one second (or three frames) with 2/3 overlap (see Figure 6). We did not use a high-pass filter of 10 Hz to mask low-frequency activities and did not manipulate the range of the Doppler as it was proposed in the paper, since this information could be crucial for distinguishing our classes (e.g., hands on the wheel while driving vs. autonomous driving). Instead, we used the IQR-range normalization where values exceeding the 75th percentile were not considered for the Doppler-trajectory computation. In the next step, following the architecture of the best-performing classifier and the training steps (see [32]), we built a bagging classifier. The training and testing datasets were generated in two ways: splitting the data as equally as possible into ten folds and using nine of them for training, and one for validation, as proposed by [32]. Next, leave-one-participant-out cross-validation was performed to achieve a possible comparison to our method. The reported results are the average over the validation splits.

**Figure 7:**
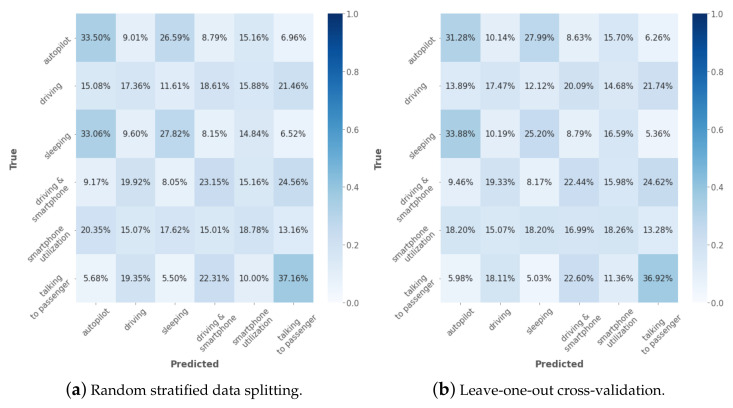
Confusion matrices of the obtained classification results using Ensemble classifier ([32]).

Figure 7a,b represent the obtained confusion matrices for the classification performance using the ensemble classifier of [32]. Using random stratified data splitting for training and testing, the model achieved an average classification accuracy of 26.3% over six classes. The highest classification accuracy of 37.16% was observed for the class *talking to passenger*. *Autopilot* was the second best predicted class with an accuracy of 33.50%. The classification accuracy for the four remaining classes was between 0.69 and 11.15 percentage points over the level of random guessing at 16.67%. A slightly lower classification accuracy but a similar classification pattern was observed after applying leave-one-participant-out cross-validation. The highest classification accuracy of 36.92% was observed for the class *talking to passenger*, which was followed by class *smartphone utilization*. The remaining classes were either slightly over or under the level of random guessing. The obtained performance drastically deviates from the one reported in the original work of [32]. The low classification performance on the *RaDA* dataset can be explained in several ways. First, fundamental differences exist between our UWB radar and the FMCW radar used in the original study. Secondly, the higher sampling rate used in [32] could bear a larger amount of available data for model training. In our work, the minimal size of the window was constrained by the frame rate of the used radar. Next, the proposed method did not explicitly consider possible outliers in the data while focusing on the high-frequency components. Finally, in the original work, their classes considered primarily hand-crafted features including head position and rotation (that were class-differentiating), while our data also include scattering information from the torso. Taking these results together, the proposed method of [32] did not perform well on our data.

### 5.2. ResNet-18

We trained a standard PyTorch implementation of ResNet-18 with weights pre-trained on ImageNet-1K. The stochastic gradient descent (SGD) optimizer with a momentum of 0.9 was used. To decrease the training time, we used the One Cycle Learning Rate scheduler [50]. This method is based on the phenomena of “super-convergence” which can be observed when training with the one-cycle learning rate schedule. Furthermore, the larger possible maximum learning rate can result in an additional increase in classification performance. The maximum learning rate was set to 0.01. The initial learning rate was chosen to be one-tenth of the maximum learning rate. We used a mini-batch size of 40 and trained for 20 epochs. A higher number of epochs did not lead to any significant improvement in classification performance. For the training and validation, the input data were repeated three times in the channel dimension and resized to 224 × 224 pixels.

### 5.3. LSTM

We built a uni-directional LSTM model. The number of features in the hidden state was set to 6, and the number of recurrent layers was 2. The learning rate of 0.001 was used. To prevent overfitting, a dropout layer with a 20% dropout rate was used. The mini-batch size was set to 8, and the number of epochs was set to 80. The number of input features was set to *1024 × 24* corresponding to the Doppler frequency range and bin range. We used the whole sequence of each action split into single frames (approximately 0.34 s per frame) for training and validation. Because of slight variations in the length of obtained recordings, all sequences were cropped to the shortest length of 163 frames (56.21 s) for the model training and evaluation. Data exceeding this range were neglected.

## 6. Results

The classification performance for the ResNet-18 architecture is reported in Table 4. The perfect average accuracy of 100% over all 6 classes was achieved with random data splitting both with and without IQR normalization. However, a drop in accuracy of 28.7 percentage points was observed for the same architecture when using leave-one-out cross-validation. Without IQR normalization, this decrease was almost 32.6 percentage points. Clearly, the random splitting leads the model to overfit strongly, which is possibly due to the prevalence of features specific to individual persons. In contrast, the models show a rather moderate result when being evaluated using cross-validation. This demonstrates the challenge of inter-person generalization of systems trained with radar data and also the challenge level of the driving monitoring application. Therefore, the models with random splitting are not considered to be the best.

The highest average classification accuracy of 71.3% was obtained for the ResNet-18 model using IQR normalization (see Figure 5a). The class *smartphone utilization* belongs to the most well-predicted classes with 91.36% accuracy, which was followed by *autopilot* with 90.93% and *talking to passenger* with 81.05%, respectively. The lowest accuracy values were observed for the classes *driving and smartphone utilization* and *driving* with 52.51% and 55.88%, respectively. Class *driving* had a high confusion with the class *driving and smartphone utilization*, whereas the latter had a high confusion with the classes *driving* and *talking to passenger*. Importantly, all of these three classes shared the same basic driving activity. In addition, the position of the right hand, as well as the intensity of the smartphone utilization was moderated by the need to maintain the proper lane and to avoid any collision, which could additionally impede the class prediction. Similarly, the confusion between the classes *sleeping* and *autopilot* can be explained. In the experimental condition, sleeping was defined as a specific head flexion for which the depth varied among participants. Taking into account that in both classes, the subject remained still in the driving chair, we assume that this confusion rate is due to the definition of the experimental class. The absence of IQR normalization leads to a decrease in the average classification accuracy to 67.4%.

For the training and evaluation of the LSTM model, we used only the leave-one-out cross-validation method. The highest average classification accuracy of 67.2% was observed using IQR normalization. The classes *talking to passenger* followed by *smartphone utilization* achieved the highest classification accuracy (see Figure 5) with 96.50% and 94.97%, respectively, which were followed by the class *driving* with 69.54% accuracy. The lowest classification accuracy was observed for the class *driving and smartphone utilization* with 35.98%. The confusion pattern between the classes *sleeping* and *autopilot*; and *driving and smartphone utilization*, *talking to passenger* and *driving* resembled those in the ResNet-18 model. The high confusion between the classes *driving and smartphone utilization* with the classes *driving* and *driving and talking to passenger* can be explained in an analogous manner as for ResNet-18. Importantly, to estimate a single class, the LSTM model received the whole one-minute sequence. Therefore, the proposed results are rather for a general model evaluation and not for a real-time driver monitoring scenario. The absence of the IQR normalization led to a drop in the classification accuracy to 43.9%. Interestingly, while in the case of ResNet-18, the use of IQR normalization led to an increase of 3.9 percentage points in classification accuracy, for the LSTM model, the difference amounted to 23.3 percentage points.

## 7. Conclusions

In this work, we presented a novel dataset with driving activities captured by a low-cost UWB radar. Our proposed driving activity recognition approach has demonstrated the feasibility of the system to recognize distracted driving behavior. It has also shown a significant improvement in classification accuracy compared to the state-of-the-art machine learning method that uses Doppler-trajectory features. Furthermore, we have evaluated different cross-validation techniques. It was demonstrated that radar-based activity recognition cannot be easily generalized to a new, unseen driver. Finally, we have shown that a simple normalization technique is able to significantly increase the classification accuracy of the deep neural networks on new, unseen drivers, especially for the LSTM model.

In the future work, a large-scale evaluation of the proposed approach under varying driving conditions and a larger number of participants could be performed. It is also important to investigate ways to reduce the ambiguity between the classes with high confusion levels, as it is vital for the correct system response in safety-critical driving scenarios.

## Figures and Tables

**Table 1:** Technical settings of Xethru X4M02 used for data recording.

Parameter	Value
Bandwidth (GHz)	7.25–10.20
Frames Per Second	50
Doppler Samples	1024
Doppler Frequency Range (Hz)	−8.5–8.5
Range Bins	24
Measurement Range (m)	0.4–1.2

**Table 2:** Overview of the data extracted from *RaDA*. Each file contains a one-second window from a particular driving action.

No.	Action	Nb. of Samples
1	Driving	1747
2	Autopilot	1844
3	Sleeping	1708
4	Driving & smartphone utilization	1692
5	Smartphone utilization	1715
6	Talking to passenger	1700
**Total size**		10,406

**Table 3:** Weight and height of participants in the *RaDA* dataset.

Participant	Height (cm)	Weight (kg)
1	188	85
2	169	50
3	178	64
4	180	93
5	178	90
6	167	74
7	172	55
8	179	77
9	170	63
10	164	59

**Table 4:** Average classification performance for driving activity recognition on the *RaDA* dataset using a re-implementation of the Ensemble classifier ([32]), ResNet-18 & LSTM. Bold: best results.

Architecture	Validation Type	IQR Norm.	Accuracy	F1-Score
Ding et al. [32]	10-fold cross-validation	✔	0.263	0.261
Ding et al. [32]	Cross-validation (Leave-one-out)	✔	0.252	0.249
ResNet-18	Random splitting (80% / 20%)	–	1.0	1.0
ResNet-18	Random splitting (80% / 20%)	✔	1.0	1.0
ResNet-18	Cross-validation (Leave-one-out)	–	0.674	0.640
ResNet-18	Cross-validation (Leave-one-out)	✔	**0.713**	**0.690**
LSTM	Cross-validation (Leave-one-out)	–	0.439	0.351
LSTM	Cross-validation (Leave-one-out)	✔	**0.672**	**0.590**

## Data Availability

Data and materials are available from the website: http://projects.dfki.uni-kl.de/rada/ (accessed on 14 November 2022).
